# Proton magnetic resonance spectroscopy (MRS) in individuals with internet gaming

**DOI:** 10.3389/fpsyt.2022.1031947

**Published:** 2022-12-22

**Authors:** Erin C. McGlade, Doug Hyun Han, Sun Mi Kim, Xianfeng Shi, Kirsten Cline, Deborah Yurgelun-Todd, Perry F. Renshaw

**Affiliations:** ^1^Huntsman Mental Health Institute, The University of Utah, Salt Lake City, UT, United States; ^2^VA Salt Lake City MIRECC, Salt Lake City, UT, United States; ^3^Department of Psychiatry, Chung Ang University Hospital, Seoul, Republic of Korea

**Keywords:** proton magnetic resonance spectroscopy, N-acetylaspartate, attention deficit hyperactivity disorder, internet gaming disorder, dorsolateral prefrontal cortex

## Abstract

**Background:**

Various comorbid psychiatric diagnoses, including attention deficit hyperactivity disorder (ADHD), have been reported in individuals with internet gaming disorder (IGD). Prior research has shown alterations in brain metabolites, including N-acetylaspartate (NAA), and combined glutamate and glutamine in patients with ADHD that were similar to those observed in patients with IGD. We hypothesized that the decreased NAA levels in the IGD group would be associated with a history of ADHD.

**Methods:**

Forty adults participated in this study. Participants were classified as having a high risk for IGD if they had a total score higher than 21 on the IGD Scale-short form. Proton magnetic resonance spectroscopy (^1^H-MRS) and high-resolution structural magnetic resonance imaging (MRI) data were acquired using a 3 Tesla Siemens Prisma scanner system.

**Results:**

Levels of NAA within the right prefrontal cortex were lower in the IGD group than those observed in the control group. In a multiple linear regression analysis, internet addiction test scores and history of ADHD were shown to predict increased game play. In addition, history of ADHD predicted lower levels of NAA within the right prefrontal cortex.

**Conclusion:**

The preliminary results of current study suggest a mediating effect of ADHD on the severity of internet game play as well as the levels of NAA within the dorsolateral prefrontal cortex (DLPFC). The inclusion of ADHD in IGD research is important and deserving of further consideration.

## 1 Introduction

In 2017, the World Health Organization (WHO) proposed the diagnostic criteria for gaming disorder (GD) and, in 2019, officially approved it (1). WHO modeled the criteria for GD on prior definitions of substance use disorders, including defining GD as a pattern of gaming that included lack of control, increased gaming despite negative consequences, and prioritizing gaming over other activities. Although several studies have supported GD as a psychiatric diagnosis (2), some scholars have expressed concern over the official inclusion of GD into the International Classification of Diseases (ICD)-11 for multiple reasons, including beliefs that the inclusion of GD in the ICD was a premature decision, concerns about over-pathologizing and stigmatizing gamers due to lack of clinical symptoms or distress, lack of overall consensus, and the high rate of comorbidities (3). In 2013, internet gaming disorder (IGD) was included in the American Psychiatric Association’s Diagnostic and Statistical Manual of Mental Disorders, 5th Edition (DSM-5) as a “condition for further study” (4). Criteria included preoccupation with gaming, withdrawal if not able to game, tolerance and need for increased time gaming, and loss or forgoing other activities due to gaming. While the ICD-10 and DSM-5 criteria for GD/IGD overlap somewhat, research has shown that GD is a stricter definition of problematic gaming, that GD may be more severe than IGD, and that GD emphasizes functional impairment more than IGD (5).

Prior research on IGD has shown a high comorbidity between IGD and psychiatric disorders, most notably attention deficit hyperactivity disorder (ADHD) (5–8). In a study on the overlap between IGD and GD, Starcevic et al. (5) suggested that ADHD was more common in patients that had both IGD and GD compared with those that had IGD alone or to a population of general gamers. Cabelguen et al. (6) found that 39% of GD patients also screened positive for ADHD. Impulsivity and low self-esteem were both predictors of comorbid ADHD and GD, and the authors suggested that problematic internet game play may be a dysfunctional way to cope with emotional dysregulation in patients with ADHD or may provide a virtual escape. Evren et al. (7) reported that the presence of probable ADHD could predict the severity of internet addiction and IGD symptoms in addition to physical aggression, anger, and hostility. In a 3-year clinical cohort study, Lee et al. (8) suggested that comorbid ADHD in IGD patients was related to poorer clinical outcomes. The recovery rates after an 8-week treatment course were 60% for individuals identified as having IGD and comorbid ADHD, compared to 93% for those having only IGD. Additionally, the authors suggested that changes in ADHD symptoms could be associated with the changes in IGD symptoms during the post-treatment follow-up period.

Just as prior research has shown significant diagnostic overlap between GD/IGD and ADHD, similar alterations in brain metabolites have been observed in patients with these psychiatric diagnoses. Using magnetic resonance spectroscopy (MRS), the studies comparing IGD and ADHD have focused on altered levels of brain metabolites such as N-acetylaspartate (NAA), and combined glutamate and glutamine (Glutamate + Glutamine) in the dorsolateral prefrontal cortex (DLPFC) and anterior cingulate brain regions. The altered metabolites in patients with ADHD were comparable to those observed in patients with IGD (9–12). The DLPFC and anterior cingulate are well known candidate brain regions in patients with ADHD and patients with IGD (13, 14). In a systemic review and meta-analysis, the reduction of gray matter (GM) volume within the DLPFC and anterior cingulate cortex was associated with problematic internet use (13). In a meta-analysis review, patients with ADHD were reported to have inadequate brain responses within the DLPFC and anterior cingulate (14).

In a meta-analysis of spectroscopy for patients with ADHD, Vidor et al. (12) reported that children with ADHD showed higher concentrations of Glutamate + Glutamine in the right medial frontal area, which they suggested may be associated with cortical thinning. The ratio of N-acetylaspartate/creatine (NAA/Cr) measured by proton magnetic resonance spectroscopy (^1^H-MRS) within the DLPFC was associated with learning problems. Additionally, in our previous research, only NAA and Glutamate + Glutamine were associated with ADHD symptoms (10). There were no specific findings of choline related to ADHD (11).

Han et al. (10) found that the level of NAA within the right frontal cortex in patients with IGD was decreased compared with the NAA levels in healthy participants. In addition, the severity of IGD symptoms was negatively correlated with the level of NAA within the right frontal cortex in patients with IGD (10). In a comparison of three groups, including patients with only ADHD, patients with ADHD + IGD, and healthy participants, patients with ADHD and patients with ADHD + IGD showed decreased levels of NAA within the right frontal cortex compared with healthy participants (9).

Based on prior research examining internet gaming and ADHD, we hypothesized that comorbid ADHD could significantly predict increased internet gaming. In addition, we hypothesized that decreased NAA levels and increased Glutamate + Glutamine levels in the higher internet gaming group might be associated with a history of ADHD.

## 2 Materials and methods

### 2.1 Participant demographics and diagnostic screening

Participants were recruited through advertisements posted around The University of Utah and in the surrounding community. The inclusion criteria were as follows: (1) age > = 18 years old and (2) intelligence quotient (IQ) > = 80. The exclusion criteria were as follows: (1) History of psychotic disorder including schizophrenia, bipolar disorder, and major depressive disorder with psychotic feature, (2) history of claustrophobia, and (3) contra indication for magnetic resonance imaging (MRI) scanning including cardiac implantable electronic device, insulin pumps, gastric reflux devices, and mental implant.

Demographic information, including age, sex, handedness, and education, was acquired using a standardized self-report demographic form. Game time in hours per week was assessed *via* two questions: “How much time (in hours) on average do you spend playing video games during a weekday? (This includes any type of games on a cell phone, computer, tablet, video game console, arcade, or internet).” and “How much time (in hours) on average do you spend playing video games during a weekend day? (This includes any type of games on a cell phone, computer, tablet, video game console, arcade, or internet).” The hours per week of weekday and weekend game play were summed to create the variable Game time (hours/week).

All participants were also screened using the Mini International Neuropsychiatric Interview (MINI) (15) to assess for comorbid psychiatric disorders. The ADHD module of the Kiddie-Sads-Present and Lifetime Version (K-SADS-PL) (16) was included to assess the history of ADHD. The Wechsler Abbreviated Scale of Intelligence (WASI) Vocabulary and Matrix Reasoning subscales (17) were administered to calculate IQ. A psychologist administered the scales and clinical measures. Intra-class correlation coefficients of MINI, K-SADS-PL, WASI were 0.91, 0.85, and 0.77, respectively.

In addition, the Internet Gaming Disorder Scale-short form (IGDS9-SF) (18), Hamilton Anxiety scale (HAM-A) (19), and Hamilton Rating Scale for Depression (HAM-D) (20) were completed to assess the clinical symptoms. Participants were classified into two groups: a high risk for IGD group (+ IGD) if they had a total score higher than 21 on the IGDS9-SF and a low risk for IGD group (−IGD) if they had a total score less than 21 (21). Through screening, forty adults participated in this study.

The University of Utah Institutional Review Board approved the research protocol for this study (approved date: 17 January, 2019, IRB number: 00117956). Written informed consent was provided by all participants.

### 2.2 Clinical scales

The HAM-A is a clinician-administered scale designed to quantify anxious symptomatology. It consists of 14 items defined by a series of symptoms, including anxious mood, tension, and fears. Intraclass correlation coefficients between 0.74 and 0.96 have been reported for this measure (22).

The HAM-D is based on the clinician’s interview with the participant and probes symptoms such as depressed mood, guilty feelings, suicide, sleep disturbances, anxiety levels, and weight loss. Research has demonstrated a validity coefficient of 0.85 (23).

IGDS9-SF consists of nine items in which a higher total score indicates a higher likelihood of IGD. Each item was assessed with a five-point Likert scale from never (1) to very often (5) (18). In an Italian population, the cut-off score of 21 for an IGD risk group was obtained (21).

### 2.3 MRS processing

**FIGURE 1:**
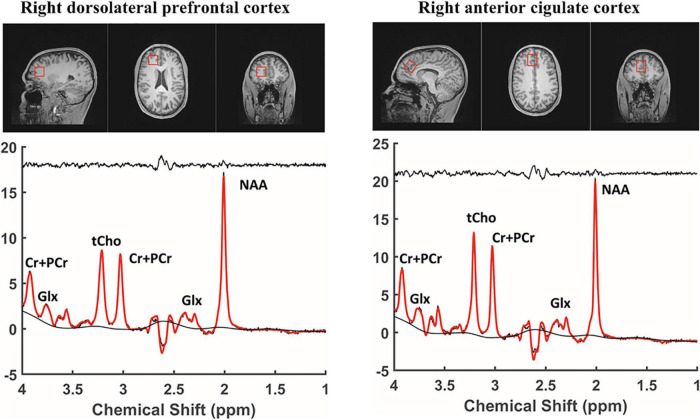
Regions of interest and MRS spectrum. MRS, proton magnetic resonance spectroscopy; NAA, N-acetyl-aspartate; Glx, Glutamate + Glutamine; tCho, total choline; Cr + PCr, Choline + phosphatidyl choline.

Structural magnetic resonance (MR) and ^1^H-MRS data were acquired using a 3 Tesla Siemens Prisma scanner system (Siemens Medical Solutions, Erlangen, Germany) with gradients (80 mT/m strength and 200 T/m/s slew rate) and a 32-channel head coil. All ^1^H spectra were acquired using a Point RESolved Spectroscopy (PRESS) with a localized single voxel pulse sequence. Voxels were sequentially placed in the right DLPFC and anterior cingulate cortex, as shown in Figure 1. Spectroscopic data from cubic volumes of 2.5 cm × 2.5 cm × 2.5 cm were obtained with a single voxel PRESS sequence of TR/TE = 2000/144 ms, a receiver bandwidth of 1 kHz, 128 averages, and a vector size of 1,024. To facilitate voxel placement, high resolution T1 weighted images were acquired using a three-dimensional magnetization-prepared rapid gradient-echo acquisition (MPRAGE) pulse sequence with the following imaging parameters: TR/TE = 2500/2.9 ms; Flip angle = 8°; Field of View = 256 mm × 256 mm × 176 mm; 256 × 256 × 176 matrix size; 1 mm × 1 mm × 1 mm spatial resolution; Bandwidth = 192 Hz/pixel. All ^1^H MRS data were fit using the commercially available Linear Combination (LC) Model software (24) in conjunction with a simulated basis set. The metabolite signal was normalized to the unsuppressed water signal. The derived metabolite concentrations were expressed in the institutional unit, defined in the LCModel manual.^1^ The LC Model fit analysis window was set to cover the 1.0–4.0 ppm chemical shift region. Figure 1 shows a representative MRS spectrum with a baseline spectrum, fitted spectrum, and residue noise spectrum. Metabolites fit with Cramer Rao lower bounds less than 20% were included in the final analysis. Brain tissue segmentation was performed using the brain extraction tool (BET) (25) and FMRIB’s automated segmentation tool (FAST) (26) provided with the freely available FMRIB software library (27). Home-built MATLAB functions were used to extract 3D volumes corresponding to the spectroscopic voxels and to compute the GM, white matter (WM), and cerebrospinal fluid (CSF) tissue content inside the voxel for each subject. The WM fraction was calculated as the ratio to total brain matter using the formula: 100 × WM/(GM + WM + CSF).

### 2.4 Statistical analysis

Demographic data, including mean age, years of education, game playing time, IGDS9-SF scores, IQ, HAM-A, HAM-D, and WASI were compared between the + IGD and −IGD groups using Mann–Whitney tests. Variables, including sex, handedness, comorbidity total, comorbidity ADHD, and comorbidity MDD were compared between the high risk for the IGD group and low risk for IGD group using Fisher’s exact tests. Metabolite levels (NAA and Glutamate + Glutamine) were also compared between the two groups using Mann–Whitney tests.

To predict increased internet game play, a multiple linear regression analysis was performed with the independent variables of age, average hours per week of game play, HAM-A, HAM-D, comorbid ADHD, comorbid MDD, and the level of NAA, and the dependent variable of IGDS9-SF total score.

To predict the level of the metabolite NAA, a multiple linear regression analysis was performed with the independent variables of age, average hours per week of game play, HAM-A, HAM-D, comorbid ADHD, comorbid MDD, and the level of metabolites, and the dependent variable of the level of NAA.

Comparison analyses of demographic, clinical, and metabolites data showed significant differences in the comorbidities of ADHD and MDD and NAA level between the high risk for the IGD group (+ IGD) and low risk for IGD group (−IGD). Those factors were therefore selected as independent variable in the linear regression model. Because there were no significant differences in the level of Glutamate + Glutamine within the right prefrontal cortex between the −IGD group and the + IGD group, a multiple linear regression analysis for predicting the level of metabolite Glutamate + Glutamine was not performed. For the same reason, a multiple linear regression analysis for predicting the level of the metabolite NAA and Glutamate + Glutamine within the anterior cingulate cortex was not performed.

The IGDS9-SF total scores between the −IGD, + IGD with ADHD, and + IGD with MDD groups were analyzed using an ANOVA. The NAA levels between the −IGD, + IGD with ADHD, and + IGD with MDD groups were also analyzed using an ANOVA. Statistical significance was set at a *p*-value of less than 0.05. All static assessments were performed using IBM SPSS 24 (IBM^®^ SPSS^^®^^ Statistics, Seoul, Republic of Korea).

## 3 Results

### 3.1 Demographic and clinical characteristics

There were no significant differences in age, sex, handedness, years of education, average weekly hours of game play, HAM-A, HAM-D, or WASI scores between the −IGD and + IGD groups. There were differences in comorbidity ratio, but these differences were not statistically significant. There were significant differences in IGDS9-SF between the −IGD and + IGD groups (Table 1).

**TABLE 1:** Demographic and psychological characteristics.

	−IGD group (*n* = 19)	+ IGD group (*n* = 21)	Statistics
Age	23.6 ± 3.2	22.4 ± 3.9	*z* = 1.19, *p* = 0.24
Sex (male/female)	15 (78.9%)/4 (21.1%)	18 (85.7%)/3 (14.3%)	χ^2^ = 0.32, *p* = 0.57
Handed (left/right)	3 (15.8%)/16 (84.2%)	2 (9.5%)/19 (90.5%)	χ^2^ = 0.36, *p* = 0.55
Education (years)	14.4 ± 4.4	13.8 ± 1.6	*z* = 1.13, *p* = 0.27
Game time (hours/week)	19.6 ± 11.0	23.4 ± 10.9	*z* = 1.13, *p* = 0.26
IGDS9-SF	13.5 ± 3.1	25.4 ± 4.6	*z* = 4.69, *p* < 0.01*
Comorbidity total	3 (15.5%)	10 (47.6%)	χ^2^ = 4.64, *p* = 0.07
ADHD	1 (5.3%)	4 (19.0%)	χ^2^ = 1.73, *p* = 0.19
MDD	2 (10.5%)	6 (28.6%)	χ^2^ = 2.03, *p* = 0.15
HAM-A	2.1 ± 3.5	3.5 ± 4.7	*z* = 0.96, *p* = 0.34
HAM-D	2.1 ± 3.1	3.6 ± 4.9	*z* = 0.95, *p* = 0.34
WASI	116.5 ± 11.3	115.9 ± 10.7	*z* = 0.29, *p* = 0.77

*Statistically significant. High risk for IGD (+ IGD) group, low risk for IGD (−IGD) group. IGDS9-SF, internet gaming disorder scale-short form; IAT, internet addiction test; ADHD, attention deficit hyperactivity disorder; MDD, major depressive disorder; HAM-A, Hamilton anxiety rating scale; HAM-D, Hamilton rating scale for depression; WASI, Wechsler abbreviated scale of intelligence.

### 3.2 Tissue composition of MRS voxels and metabolite levels in right dorsolateral prefrontal cortex and right anterior cingulate cortex

There were no significant differences in the mean volume percentage of GM and WM in the MRS voxels of the right DLPFC voxel between the two groups. The levels of NAA within the right DLPFC in the + IGD group were lower than those observed in the −IGD group. There was no significant difference in the level of Glutamate + Glutamine within the right DLPFC between the two groups (Table 2).

**TABLE 2:** Metabolism in the dorsolateral prefrontal cortex and anterior cingulate cortex.

	−IGD group (*n* = 19)	+ IGD group (*n* = 21)	Statistics
**Dorsolateral prefrontal cortex**			
Gray matter (vol %)	28.8 ± 3.5	27.8 ± 3.3	*z* = −0.93 *p* = 0.36
White matter (vol %)	67.5 ± 4.0	68.7 ± 4.1	*z* = −0.81 *p* = 0.42
NAA_DLPFC*	24.1 ± 3.6	20.7 ± 5.1	*z* = 2.21 *p* = 0.03
GluGln_DLPFC	14.8 ± 2.1	13.6 ± 3.2	*z* = 0.93 *p* = 0.35
**Anterior cingulate cortex**			
Gray matter (vol %)	60.2 ± 3.2	59.7 ± 3.0	*z* = −0.81 *p* = 0.42
White matter (vol %)	24.0 ± 2.8	24.8 ± 3.2	*z* = −0.98 *p* = 0.33
NAA_ACC	21.9 ± 4.1	20.4 ± 1.9	*z* = 1.12 *p* = 0.26
GluGln_ACC	16.5 ± 2.4	16.1 ± 1.5	*z* = 0.36 *p* = 0.75

High risk for IGD (+ IGD) group, low risk for IGD (−IGD) group. NAA, N-acetyl-aspartate; GluGln, Glutamate + Glutamine; DLPFC, dorsolateral prefrontal cortex; ACC, anterior cingulate cortex. *Statistical significance.

There were no significant differences in the mean volume percentage of GM and WM in the MRS voxels of the right anterior cingulate cortex voxel between the two groups. There also were no significant differences in the levels of NAA and Glutamate + Glutamine within the right anterior cingulate cortex between the two groups (Table 2).

### 3.3 Predictive factor for game play and the level of NAA

History of ADHD predicted increased game play in a multiple linear regression analysis (Table 3). In addition, a history of ADHD predicted lower levels of NAA within the right DLPFC (Table 3).

**TABLE 3:** Multiple linear regression analyses.

	β	*t*	*p*	95% confidential interval	
**The effects of comorbidity and the levels of NAA on the severity of IGD^1^**					
Com-ADHD*	0.570	4.452	<0.001	5.776	15.497
Com-MDD	0.086	0.685	0.498	–2.622	5.283
NAA_DLPFC	0.075	0.550	0.586	–0.265	0.461
**The effects of comorbidity and the severity of IGD on the levels of NAA^2^**					
Com-ADHD*	–0.466	–2.452	0.020	–12.103	–1.125
Com-MDD	–0.193	–1.221	0.231	–6.046	1.512
IGDS9-SF	0.122	0.550	0.586	–0.250	0.435

^1^Dependent variable: internet gaming disorder scale-short form (IGDS9-SF total score). ^2^Dependent variable: N-acetyl-aspartate within dorsolateral prefrontal cortex (NAA_DLPFC).

*Statistically significant. Com-ADHD, comorbidity of attention deficit hyperactivity disorder; Com-MDD, comorbidity of major depressive disorder.

**FIGURE 2:**
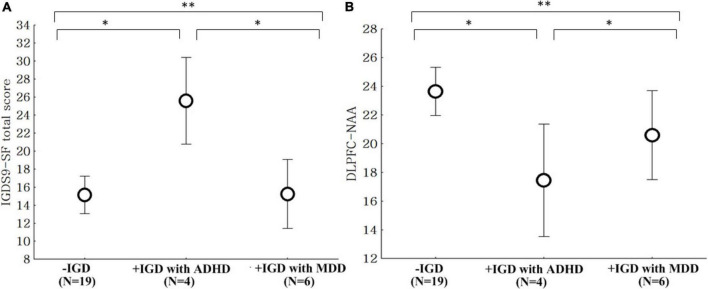
Comparison of the IGDS9-SF scores and NAA level. IGDS9-SF, internet gaming disorder scale-short; NAA, N-acetyl-aspartate. **(A)** Comparison of the IGDS9-SF total scores between the low risk for internet gaming disorder group (–IGD), high risk for internet gaming disorder with attention deficit hyperactivity disorder group (+ IGD with ADHD), and high risk group for internet gaming disorder with major depressive disorder group (+ IGD with MDD). ^**^ANOVA, *F* = 8.42, *p* = 0.001, *post hoc: *p* < 0.05, –IGD and + IGP with MDD < + IGP with ADHD. **(B)** Comparison of the NAA level within the dorsolateral prefrontal cortex (DLPFC) between the low risk for internet gaming disorder group (–IGD), high risk for internet gaming disorder with attention deficit hyperactivity disorder group (+ IGD with ADHD), and high risk for internet gaming disorder with major depressive disorder group (+ IGD with MDD). ^**^ANOVA, *F* = 5.06, *p* = 0.011, *post hoc: *p* < 0.05, –IGD and + IGP with MDD > + IGP with ADHD.

The IGDS9-SF scores in the + IGD with ADHD group were higher than those observed in the −IGD and + IGD with MDD groups (Figure 2). The levels of NAA in the + IGD with ADHD group were lower than those observed in −IGD and + IGD with MDD groups (Figure 2).

## 4 Discussion

The current results show that the levels of NAA within the right DLPFC of the + IGD group were lower than those observed in −IGD group. A history of ADHD diagnosis was associated with increased game play and decreased levels of NAA within the right DLPFC.

### 4.1 Metabolite levels in the right dorsolateral prefrontal cortex and right anterior cingulate cortex in the higher internet gaming group

The current study also showed that NAA levels within the right DLPFC were lower in the + IGD group compared with the −IGD group. There was no significant difference in the level of Glutamate + Glutamine within the right DLPFC between the two groups. There were also no significant differences in the levels of NAA and Glutamate + Glutamine within the right anterior cingulate gyrus between the two groups.

The findings of increased levels of NAA within the prefrontal cortex in the + IGD group were consistent with previous studies (9, 10). In a previous MRS study, the levels of NAA within the right frontal cortex in patients with IGD were lower than those observed in healthy participants (10). It has been proposed that levels of NAA are associated with energy production, which may reflect changes in brain activity (28, 29). Taken together, the evidence of decreased levels of NAA within the DLPFC suggests that increased internet game play is associated with hypofrontality.

This study found no alteration in the Glutamate + Glutamine levels, while Bae et al. (9) reported decreased Glutamate + Glutamine levels in the IGD group. Based on these findings, Bae et al. speculated that a decreased level of Glutamate + Glutamine may be associated with increased dopamine related to pathologically excessive internet gaming play (30, 31). The inconsistent findings between this study and previous studies may be due to the number of participants enrolled in the studies and the participant’s clinical characteristics. This study recruited 40 adult general game users in the United States who reported playing internet games for an average of 20 h per week while the studies by Bae et al. included 97 adolescents from Republic of Korea who were enrolled in a hospital treatment program for problematic internet gaming and reported playing more than 35 h per week (9, 10).

Glutamine and glutamate are thought to be associated with neural pruning and neurotrophic activity, which are increased in adolescents with ADHD (12). The participants in the previous study were adolescents, including ADHD patients (10), while the participants in this study were healthy adults. The age and pathologic ADHD condition may lead to inconsistent findings between the two studies. Additionally, the differences in participants could be linked to the novel findings in this study. The comorbidity of ADHD could also affect the severity of problematic internet gaming in the general group through similar brain dysfunctions within the prefrontal cortex, which were observed in the pathologic group.

### 4.2 The effects of comorbid ADHD on the severity of internet gaming and the levels of NAA within the dorsolateral prefrontal cortex in internet game players

In the multiple linear regression analyses of the current study, a history of ADHD was associated with IGD. The + IGD with ADHD group showed higher IGDS9-SF total scores than those observed in the + IGD without ADHD and −IGD groups. These results were similar to previous studies (32, 33). In a systemic review on the relationship between IGD and ADHD, Duller et al. (33) suggested that the symptoms of inattention seen in ADHD were more consistently associated with GD than other symptoms of ADHD, such as hyperactivity. In a survey of 108 adolescents with ADHD (96 males) and 147 healthy adolescents (114 males), Berloffa et al. (32) reported that 44% of the ADHD group and 9.5% of the healthy adolescent group scored above the cutoff, suggesting IGD. In addition, the authors suggested that the severity of inattention was significantly related to IGD (32). In a correlation study of internet addiction, IGD, and mobile phone addiction, Menendez-Garcia et al. (34) suggested that ADHD was a risk factor for developing internet addiction and IGD; however, good social adjustment could buffer that association.

In the multiple linear regression analyses of this study, a history of ADHD was associated with decreased levels of NAA within the DLPFC. The biological meaning of NAA, however, is still debated. Lower NAA level within the frontal lobe is thought to be related to lower neuronal integrity involving emotional and cognitive regulation, decision making, and impulsivity (35–37). Many studies on ADHD have reported emotional dysregulation, disturbance in decision making, and impulse control difficulties (35–37). Interestingly, the + IGD with ADHD group showed decreased levels of NAA within the DLPFC compared with the + IGD without ADHD and −IGD groups. In a comparison of the three groups, including patients with only ADHD, patients with ADHD and IGD, and healthy participants, the patients with only ADHD and patients with ADHD and IGD had the similar finding of decreased levels of NAA within the frontal cortex compared with the healthy control (9). Moreover, a meta-analysis of IGD and ADHD studies using structural and functional MRIs showed that patients with IGD and patients with ADHD shared structural and functional alterations within the prefrontal cortex (38).

### 4.3 Limitations and future directions

There were several limitations to this study. First, the number of participants was modest, which is challenging for generalizing the results. Importantly, the current participants included a community sample, which could have key differences compared with other samples, which are often individuals hospitalized various disorders, including IGD. Additionally, the data in this study were cross-sectional and included only adult participants. Given the classification of ADHD as a neurodevelopmental disorder and brain changes associated with both ADHD and IGD, future directions include examining clinical symptoms and brain changes over time and throughout development. Our sample was likely healthier overall compared with those recruited from hospitals. Additionally, the data on internet game play were all self-reported, which could be associated with biases, including social desirability and short-term recall. Future research may benefit from the behavioral tracking of game play, including through the internet and mobile phones. Lastly, this study focused on IGD with ADHD and could be associated with the severity of game play and associations between IGD and ADHD and NAA. Future studies could also include a non-gaming control group with ADHD to understand better the association between ADHD and NAA in the absence of IGD.

## 5 Conclusion

These preliminary results of current study suggest the crucial effect of ADHD on the severity of internet game play. Moreover, decreased levels of NAA in participants with ADHD and at high risk for IGD were observed, suggesting that individuals with comorbid ADHD and at high risk for IGD may have metabolite alterations that differ from individuals at low risk for IGD and from individuals who are at high risk for IGD but not ADHD. The inclusion of ADHD in IGD research is important and deserving of further consideration.

## Data availability statement

The original contributions presented in this study are included in the article/supplementary material, further inquiries can be directed to the corresponding author.

## Ethics statement

The studies involving human participants were reviewed and approved by The University of Utah Institutional Review Board. The patients/participants provided their written informed consent to participate in this study.

## Author contributions

EM and KC contributed to patients’ recruitment, data collection, and processing. XS, SK, and DH analyzed the data. EM, DY-T, DH, and PR participated in drawing up the manuscript and were involved to the intellectual workup for the article. All authors read and approved the final manuscript.

## Footnotes

1
www.s-provencher.com/pub/LCModel/manual/manual.pdf
